# Exploring the Benefits of Virtual Reality-Assisted Therapy Following Cognitive-Behavioral Therapy for Auditory Hallucinations in Patients with Treatment-Resistant Schizophrenia: A Proof of Concept

**DOI:** 10.3390/jcm9103169

**Published:** 2020-09-30

**Authors:** Laura Dellazizzo, Stéphane Potvin, Kingsada Phraxayavong, Alexandre Dumais

**Affiliations:** 1 Research Center of the Institut Universitaire en Santé Mentale de Montréal, 7331 Hochelaga, Montreal, QC H1N 3V2, Canada; laura.dellazizzo@umontreal.ca (L.D.); stephane.potvin@umontreal.ca (S.P.); 2 Department of Psychiatry and Addictology, Faculty of Medicine, University of Montreal, Montreal, QC H3T 1J4, Canada; 3 Services et Recherches Psychiatriques AD, Montreal, QC, Canada; kingsada.phraxayavong@gmail.com; 4 Institut National de Psychiatrie légale Philippe-Pinel, Montreal, QC H1C 1H1, Canada

**Keywords:** treatment resistant schizophrenia, auditory verbal hallucinations, cognitive behavioral therapy, avatar, virtual reality

## Abstract

Background: Combining cognitive behavioral therapy (CBT) for psychosis with another psychosocial intervention comprising virtual reality (VR)-assisted therapy (VRT) may improve targeted outcomes in treatment-resistant schizophrenia patients. Methods: Ten participants having followed CBT were part of our comparative clinical trial comparing VRT to CBT and were selected at the end of the study as they desired to continue to achieve improvements with VRT (CBT + VRT). Clinical assessments were administered before/after treatments and at follow-ups. Changes in outcomes were examined using linear mixed-effects models. To gain a more in depth understanding on CBT + VRT, therapists’ notes, and open interviews on a sub-group of patients were qualitatively analyzed. Results: Findings showed that the sequence of both interventions was appreciated by all patients. Several significant improvements were found throughout time points on auditory verbal hallucinations, beliefs about voices, depressive symptoms, symptoms of schizophrenia and quality of life. Although most of these improvements were in similar range to those observed in our comparative trial, effects of CBT + VRT on depressive symptoms and symptoms of schizophrenia were larger than those found for either intervention alone. Conclusion: This proof of concept is the first to merge gold-standard CBT with VRT for treatment refractory voices and to suggest a certain synergistic effect.

## 1. Introduction

Schizophrenia, especially treatment-resistant schizophrenia (TRS), is a complex, severe and disabling psychiatric disorder, which poses a significant therapeutic challenge and current treatments show limited efficacy [1]. Notably, TRS contributes to a significant loss in patients’ quality of life and is associated with a high economic burden [2]. Among the variety of debilitating symptoms, auditory verbal hallucinations (AVH) are the most reported form of hallucination [3]. Medication is generally helpful to treat these symptoms, however, up to 50% of patients are resistant to first-line antipsychotic treatments (TRS) and will continue to suffer from persistent AVH [1,4,5]. While clozapine is an effective second-generation antipsychotic reserved for TRS, as many as 40% will also fail to respond to this molecule [6,7]. It is now widely acknowledged that pharmacotherapy alone is rarely sufficient to achieve best outcomes, and that combined pharmacological and psychosocial approaches are necessary to improve long-term outcomes in those with schizophrenia [8]. Cognitive-behavioral therapy for psychosis (CBTp) has been the psychotherapy most largely endorsed and recommended by treatment guidelines [9,10,11]. Yet, at best, only moderate effects may be achieved [12,13,14,15,16,17]. Moreover, approximately 50% of patients do not respond to this approach [18]. Given the limited benefits of existing “gold-standard” psychosocial treatments for TRS, there is consequently space for therapy improvement [19]. 

New developments in cognitive-behavioral approaches for schizophrenia include the delivery of CBTp in combination with another empirically recognized psychosocial intervention with the goal of enhancing targeted treatment outcomes [20]. Offering such merges has the advantage of focusing on more than one therapeutic goal at once and may therefore better generalize in other aspects of patients’ lives [21]. These additional add-on interventions for voices should further concentrate on processes specific to voice hearing [22], such as the interpersonal and emotional dimensions of voices, and should include experiential elements embedded within the therapy. The emergence of virtual reality (VR) in psychotherapy may answer this need. This tool has shown several advantages that are noteworthy for optimizing psychotherapies including its versatility, high level of control over exposure parameters, acceptability as well as adherence to treatment [23,24,25,26]. Approaches using VR are commonly based on traditional cognitive-behavioral techniques and, in addition, due to their more experiential component, they may raise the likelihood of transferring learnings during VR sessions to patients’ everyday life [26]. VR may be helpful to allow patients to re-experience or relive moments and to explore difficult events that cannot be easily nor ethically explored in real-life to learn how to respond/behave in specific circumstances [27]. The use of VR is especially useful in the case of schizophrenia for the treatment of AVH since simple exposure is challenging due to the invisible component of voices. It is consequently possible to enhance current psychological approaches by allowing patients to be directly exposed in a personalized manner to their anxiety-inducing voices via VR. 

AVATAR therapy (AT) and VR-assisted therapy (VRT) are experiential and exposure-based interventions that permit the establishment of an intimate dialogue with patients’ voice by means of an avatar controlled in real time by a therapist. Patients can therefore learn ways to regulate their negative emotions elicited by the persecutory voice, to be more assertive and to strengthen their sense of self. The results of the two pilot trials comparing AT/VRT to treatment-as-usual [28,29] as well as a larger RCT comparing AT to supportive counseling [30] showed large effects of VR therapies on AVH in short-term follow-ups and up to a 24-week follow-up. Recently, our one-year comparative trial comparing VRT to CBT for voices showed that both interventions produced significant improvements in AVH severity and depressive symptoms. VRT did achieve, however, larger effects particularly on overall AVH. VRT also showed significant results on persecutory beliefs, quality of life and affective symptoms. Effects were also maintained up to the one-year follow-up. VRT highlights the future of patient-tailored approaches that integrates several relevant processes to potentially improve the effectiveness of generic CBT for voices. Keeping this in mind, CBT and VRT address different needs patients may have. To reduce distress, CBT for voices enables patients to normalize their psychotic experience while explicitly identifying their distorted beliefs [31,32,33]. Such approach provides patients with tools to modify these beliefs and learn to better cope with their symptoms. On the other hand, VRT is among a newer wave of dialogical approaches that aim to ameliorate the voice-hearer relationship by encouraging assertive interactions with voices and by negotiating new ways of relating. VRT focusses on repetitive practice in real-time in an emotional-inducing VR environment where patients may dialogue with their distressing voices [28,29,30,34,35]. Although these two therapies overlap in common practice, to our knowledge, no one has integrated both these models to draw on their strengths. By combining both low-intensity therapies, we hope to address issues that are particularly challenging in TRS patients. Hence, there may be benefits for patients to have gained a set of skills and knowledge with CBT that may then be experientially applied in VRT.

The aim of this proof of concept paper is therefore to detail the benefits of combining CBT for voices followed by VRT (CBT + VRT) as part of our trial comparing the efficacy of VRT to CBT. In this exploratory study, we attempt to investigate the synergistic effects of both approaches in TRS patients on our primary outcome consisting of AVH and our secondary outcomes comprising beliefs about voices, psychiatric symptoms, and quality of life. Moreover, we gain insight into patients’ perspectives of the individual therapies and the combination of both approaches.

## 2. Methods

### 2.1. Participants

Participants were part of our larger comparative clinical trial comparing VRT to CBT for voices (identifier number on Clinicaltrials.gov: NCT03585127). Particularly for this study, 10 participants assigned to the CBT arm and having done their corresponding follow-up assessment requested whether they could continue to achieve improvements by following VRT as well. Patients (≥18 years old) with refractory AVH and schizophrenia or schizoaffective disorder were recruited from the Institut Universitaire en Santé Mentale de Montréal (where the therapy was provided) as well as from the community. Patients were recruited if they had been hearing persecutory voices and did not respond to at least two antipsychotic trials. All patients continued to receive standard psychiatric care and agreed to withhold from changing existing antipsychotic use over the duration of the therapy sessions. Participants were excluded if they presented a neurological disorder, an unstable and serious physical illness, or a substance use disorder in the past 12 months and if they followed another CBTp in the past year (other than the one offered by our team). The trial was conducted in accordance with the Declaration of Helsinki and was approved by the institutional ethical committee (CER IPPM 16-17-06). Written informed consent was obtained from all patients.

### 2.2. Cognitive-Behavioral Therapy for Auditory Verbal Hallucinations

Patients first began by following CBT for AVH, which consisted of nine individual and weekly sessions of one hour. These sessions were administered in an individual format by a licensed psychologist trained in CBT by Dr. O’Connor who had trained 35 psychologists throughout his career [36,37,38,39,40,41,42,43]. The CBT program was derived and adapted from current evidence-based treatments for AVH. This therapy involved a succession of learning modules as well as voice journals (task assignments) and has been previously described elsewhere [44,45]. The first three sessions consisted of patients’ anamnesis to set goals and learning about AVH. More particularly, in line with the cognitive model of hallucinations, voices were understood as being triggered rather than being related to the beliefs they held. Patients completed voice journals to reflect on their positive symptoms and associated triggers. The following three sessions focused on metacognition. In session 4, patients first learned about diverse attributional mechanisms and completed another voice journal to detect the beliefs that were the cause of their ill-being. In sessions 5 and 6, patients were taught to better interpret situations with the use of vignettes. The following two sessions were based on mindfulness exercises and they were encouraged to ask for feedback and to learn to observe. Patients learned to put forward alternative explanations to their most common beliefs about their hallucinations. Lastly, the therapy ended with a summary of learnings and relapse prevention. 

### 2.3. Virtual Reality (VR)-Assisted Therapy

The therapy was delivered by an experienced clinician (AD) who has around seven years of experience as a psychiatrist and treated over one thousand patients with major psychiatric disorders including schizophrenia [46,47,48,49,50,51,52,53,54,55]. The therapy consisted on average of nine-weekly sessions. VRT has been previously described elsewhere [29,44,45]. Briefly, patients were first requested to create and personalize the face and voice of an avatar best resembling the person or entity believed to be the source of their most distressing voice. The following therapeutic sessions consisted of three phases. Pre-immersion: the therapist discussed the preceding week and determined the objective of the therapy session with the patients. Immersion: patients were immersed in the VR environment and encouraged to enter in a dialogue with their avatar animated in real time by the therapist. Post-immersion: the therapist debriefed the patient and evaluated their feelings of their immersive experience. In the first immersive sessions, the therapist would confront patients via the avatar by verbalizing personalized distressing utterances and encouraged patients to use their usual coping strategies. The avatar’s interaction with the patient became gradually less hostile and more encouraging as sessions of VRT progressed. The patient generally became more empowered in the interaction they held with their avatar as the former developed more assertiveness and a higher self-esteem. In the final consolidation sessions, patients had the opportunity to apply what they had previously learned in the experiential sessions and to follow up on their initial objectives. Each therapy session was audio recorded and the recording was offered to patients.

### 2.4. Clinical Assessments

Clinical assessments were administered before and after each intervention and at follow-up periods by trained research psychiatric nurses. Follow-up periods for CBT could have been three, six or 12 months depending on when patients desired to undertake VRT. As for VRT, follow-up period was conducted at three months. 

The primary outcome covered the overall severity of AVH as measured with the Psychotic Symptoms Rating Scale (PSYRATS-AH) [56]. This scale comprises 11 items evaluated by interview, which was divided into four factors (distress, frequency, attribution, and loudness). Secondary outcomes included beliefs about voices, general psychiatric symptoms, and quality of life. Patients’ beliefs about their voices as well as the manner they cope with them were measured with the Beliefs About Voices Questionnaire-Revised (BAVQ-R) [57]. The BAVQ-R supports four subscales [58]: two subscales relating to beliefs (persecutory beliefs and benevolence) in addition to two further subscales that measure responses to the voices (resistance and engagement). Depressive symptoms were assessed with the Beck Depression Inventory-II (BDI-II) [59], which consists of a 21-item self-report inventory. Symptoms of schizophrenia were evaluated with the Positive And Negative Syndrome Scale (PANSS) [60]. This scale was separated into five symptom clusters (positive symptoms, negative symptoms, cognitive symptoms, hostility and excitement symptoms, and affective symptoms) [61]. Life satisfaction was evaluated with the Quality of Life Enjoyment and Satisfaction Questionnaire-Short Form (Q-LES-Q-SF) [62,63], which consists of a self-report scale of 14 items.

To gain a more in depth understanding on the combination of both therapies, we qualitatively examined the therapists’ notes of each patient in addition to interviews that were held with a sub-group of patients. For the latter part, six participants who desired to give their opinion were interviewed after having followed both interventions. These interviews were open interviews with a loose guide provided to explore topics of interest. The central questions consisted on their perspectives on each individual therapy and their combination. When appropriate, the evaluator prompted patients to expand on interesting responses. Patients’ evolution of their voices in addition to their quality of life were also assessed. These interviews were recorded and elements pertaining to the main topics of interest were transcribed. French excerpts were then translated into English.

### 2.5. Analyses

Statistical analyses were performed with SPSS Statistics for Windows (Version 25, IBM, Armonk, NY, United States). Descriptive statistics were conducted on baseline data. Changes in reported outcomes during the assessment periods were assessed using a linear mixed-effects model with maximum-likelihood estimations for missing data. Time points were defined as follows: T1 = baseline CBT, T2 = post-CBT, T3 = follow-up CBT/baseline VRT, T4 = post-VRT and T5 = three-month follow-up VRT. Since the follow-ups for the CBT arm differed between patients, we took the overall mean of follow-up periods as T3 value. The statistical threshold for significance was set at *p*-value < 0.05. Effect sizes were categorized as small (0.2), medium (0.5) and large effects (>0.8) [64]. 

As for qualitative data, the therapists’ notes and patient verbatim were annotated for any elements pertaining to changes in symptomatology, self-reflections, and overall comments on individual therapies in addition to their combination. As many headings as necessary were systematically written down to describe all aspects of content. The headings were collected, categorized into related concepts, and grouped under higher order themes to develop an integrated representation of data. Higher order themes were in line with key therapeutic processes of the interventions. For CBT, these themes were based on the topics discussed during the intervention (e.g., normalization of voices, coping strategies) and for VRT, the themes were based on prior qualitative analyses (e.g., emotion regulation, self-perceptions) [34,35]. 

## 3. Results

### 3.1. Sample Characteristics

Sample characteristics are found in Table 1. Overall, there was a greater proportion of men (80%), all were Caucasian, and most were single (90%) with a mean age of 43.4 years (Standard deviation (SD) = 14.6). The mean duration of schooling was 11.2 years (SD = 2.7). Most patients held a diagnosis of schizophrenia (80%) and the rest (20%) held a diagnosis of schizoaffective disorder. The mean duration of illness was of 16.4 years (SD = 10.9). All patients were treated with atypical antipsychotics with over two thirds of participants (70%) being prescribed clozapine. There was a mean delay of 6.8 months between the end of CBT and start of VRT. 

### 3.2. Treatment Efficacy

Table 2 summarizes the results from time points and associated effect sizes of measured outcomes.

As for the primary outcome, there was no statistically significant effect for the CBT portion (T1 to T3) for the severity of AVH as assessed with the PSYRATS-AH. Effects were of moderate magnitude (d = 0.427). Effects were statistically significant for the VRT portion (T3 to T5) for overall AVH, with most significant reduction found for AVH frequency (*p* = 0.008). The effect for the severity of AVH was additionally of moderate magnitude (d = 0.606). There were statistically significant reductions throughout overall time points for the combination on CBT + VRT treatment on the severity of AVH (*p* = 0.015), AVH frequency (*p* = 0.010), attribution (*p* = 0.019) and loudness (*p* = 0.047). A trend towards significance was observed for AVH distress (*p* < 0.1). Effects were notably significant between baseline CBT and follow-up VRT for all AVH outcomes. The effects of CBT + VRT on AVH were large (PSYRATS-AH-Total score d = 1.043; PSYRATS-AH-Distress d = 0.898; PSYRATS-AH-Frequency d = 0.859; PSYRATS-AH-Attribution d = 1.020; PSYRATS-AH-Loudness d = 0.946). There were four patients considered as treatment responders to CBT + VRT, which was defined as a decline of at least 20% on the total score of the PSYRATS-AH.

Concerning beliefs about voices measured with the BAVQ-R, there was no significant pre–post-treatment difference on overall beliefs for the CBT portion with the effect size being of small magnitude. A trend was, however, found for pre- to follow-up CBT (*p* = 0.089), yielding to a moderate effect size (d = 0.408). As for sub-scales, there was a significant pre–post reduction on persecutory beliefs (*p* = 0.049) and a significant pre- to follow-up reduction in engagement (*p* = 0.001) for CBT. Regarding the latter outcome, there was then a significant increase in engagement between pre–post as well as pre- to follow-up VRT (*p* = 0.010 and *p* = 0.028, respectively). The merge CBT + VRT showed a significant diminishment in overall beliefs about voices between baseline CBT to post VRT (*p* = 0.037) yielding to a moderate effect size between these time points (d = 0.461). Beliefs about voices then increased towards baseline value at follow-up VRT (T5). 

Depressive symptoms measured with the BDI-II significantly diminished throughout time (*p* = 0.001). There were pre–post and pre- to follow-up reduction for CBT (*p* = 0.002 and *p* = 0.030, respectively) yielding to a large effect size post treatment (d = 0.806) and a medium effect size at follow-up (d = 0.467). Depressive symptoms additionally significantly diminished for the VRT portion with a significant pre- to follow-up reduction being observed (*p* = 0.015). The merged therapies showed a significant diminishment between baseline CBT to post- (*p* = 0.004) and follow-up (*p* < 0.001) VRT with the effect being of large magnitude (d = 1.020). 

Although CBT showed no significant differences for overall symptoms of schizophrenia as measured with the PANSS, the VRT portion did yield to significant pre–post-treatment reductions (*p* = 0.003). A trend was also observed between pre-VRT and follow-up VRT (*p* = 0.097). As for the sub-scales, significant findings were found for pre-post VRT on negative symptoms (*p* = 0.024) and excited/hostility symptoms (*p* = 0.009); a trend was also observed for positive symptoms (*p* < 0.1). Overall, general symptoms significantly diminished throughout all time points (*p* = 0.013). The effect size for overall symptoms from baseline CBT to follow-up VRT was of large magnitude (d = 0.806). Moreover, there were principally statistically significant effects from baseline CBT to post VRT for most subscales. There were also significant effects of CBT + VRT throughout time points for disorganized (*p* = 0.022) and excited/hostility (*p* = 0.011) symptoms and a trend for positive symptoms (*p* < 0.1). 

Lastly, CBT showed no significant effects on quality of life. The VRT portion did reach significance from pre-VRT to follow-up VRT (*p* = 0.045) yielding to a moderate effect size (d = 0.451). In addition, there was a trend towards significance for quality of life between baseline CBT to follow-up VRT (*p* < 0.1). There was an overall small effect for quality of life from baseline CBT to follow-up VRT (d = 0.331). 

### 3.3. Qualitative Perspectives on Interventions

To complement and go beyond the statistical findings, the therapists’ notes and patient interviews found relevant elements that emerged for each therapy and their merge. First, CBT for voices was found to help patients gain better awareness into their illness and talk about their experience to better accept their diagnosis and themselves. For some, this was the first time they had the opportunity to discuss their experience in a non-judgmental environment. Patients noted that the therapist dived deep “into (their) pride,” “allowed to shed light in the fog” and “put words on blurry things.” The therapy therefore helped to clarify their experience. A large portion of CBT was aimed towards learning new strategies such as relaxation and mindfulness to diminish the breadth of voices and better manage/control voices and stressful periods when they arose. Hence, many patients verbalized being able to better manage their emotions and they felt more confident. 

Second, similarities were found with VRT. The therapy helped patients better accept themselves and their voices, while also learning to better manage their emotions. Patients noted that VRT helped to embody their voice and make their experience come to life by enabling a direct discussion with their voice (e.g., “the avatar was truly there” and they “had to face it”). A patient stated that “putting a face to the voice” was one of their favorite parts of the therapy. The therapy helped them not only improve their dialogue with their voices (i.e., self-assertiveness), but these gains also extended to their overall life by making them more open with others, thereby improving interpersonal relations. VRT allowed many patients to forgive themselves and accept past events that occurred in their life (sexual abuse, for instance). Although the initial confrontation sessions were emotionally difficult, several patients noted a feeling of liberation following therapy sessions. One patient even verbalized that the therapy enabled him/her to “chase away the demon inside (him/her)”. Lastly, patients discussed their hopes about their voices and life. For some, VRT helped make them ready to undertake new life plans, such as returning to school and work. 

Third, when looking back into both therapies, patients expressed their complementarity and found the sequence to be the best option. As noted, CBT was axed on “questioning” and “comprehension”, while VRT was axed on “taking action” and “dialogue”. CBT allowed patients to obtain the necessary tools to be able to then apply them into VRT in a more direct manner. Accordingly, one patient stated that “in retrospect, both therapies were necessary and complementary; the sequence was ideal because CBT helped to prepare the tools needed for VRT, and mostly helped with the difficult confrontation sessions of VRT.” 

## 4. Discussion 

With the limited efficacy of CBTp, it has become more established that the combination of gold-standard CBT with another empirically based psychotherapy may be valuable to address the needs of patients with schizophrenia [65], and more so those with treatment resistance. This proof of concept study aimed to explore the usefulness and effectiveness of combining CBT for voices and VRT in patients with TRS. The sequence of both low-intensity interventions was found to be appreciated by all patients. CBT + VRT was beneficial for patients with several significant improvements being found. Although most of these improvements were in similar range to those observed in our comparative trial [45], effects on certain symptoms such as depressive symptoms were larger than those found for either intervention alone. 

Concerning our primary outcome, results on CBT + VRT showed that there were large reductions from baseline to three-month follow-up treatment on overall AVH severity (d = 1.043), including distress, frequency, attribution, and loudness. Moreover, CBT + VRT achieved large effects in line with the effects reported in our comparative trial [45] and prior trials on VR therapies for AVH [28,29,30]. The CBT portion yielded to moderate effects (d = 0.427). These results are in line with our hypothesis stating that CBT + VRT may achieve greater efficacy on AVH in comparison to the small-to-moderate effects that have been observed in literature on generic CBTp [66]. Results were more mitigated on beliefs about voices as observed in our comparative trial [45]. Although not significant, there was a trend towards significance for overall beliefs about voices for CBT and the effect was of moderate magnitude (d = 0.408). More precisely, in line with the therapeutic targets of CBT, the intervention decreased persecutory beliefs as well as engagement. Engagement then increased with VRT. This is in accordance with CBT aimed at changing beliefs about voices and learning non-relational coping strategies [67] and VRT aimed at increasing a dialogue with voices. In accord to our comparative trial [45], the effect of CBT + VRT was at best of moderate magnitude. Moreover, although not statistically significant for overall time points, there was an effect on quality of life mostly after having followed VRT. This is consistent with our previous trials showing improvement in quality of life with VRT [29,45] and findings demonstrating that CBT does not generally improve quality of life [68].

Of interest, there were notable improvements observed for depressive symptoms (d = 1.020) and overall symptomatology of schizophrenia (d = 0.806), with most effects for excited/hostility and disorganized symptoms. These effects were larger than those observed in our comparative trial for either of the interventions [45]. Moreover, there were additional benefits to the combination of both interventions with significant findings being found for positive symptoms, negative symptoms, and disorganized symptoms. This is suggestive of a certain synergistic effect of combining both interventions. Although results are not clear-cut, such synergistic effects of combining both approaches were verbalized by patients as well. These findings are expected given the emphasis of CBT on learning to normalize the psychotic experience and gain new skills to manage stress and emotions in combination with VRT that then enables patients to experience strong emotions (e.g., anxiety, fear, and anger) during the dialogue with their voices to directly learn to regulate them. 

Our exploratory paper is the first to combine gold-standard CBT with VRT for the treatment of refractory AVH in patients with schizophrenia and has shown some preliminary evidence for a synergistic effect of CBT + VRT mainly on depressive symptoms (BDI) and symptoms of schizophrenia (PANSS). This proof of concept study highlights the possible benefits of having gained knowledge on the psychotic experience and acquired a set of skills with CBT that may then be applied in a more experiential manner within emotion inducing VRT. Nevertheless, this study has limitations that should be acknowledged. First, the principal limitation consists the small sample size that limits the generalization of results. A larger fully powered study would be necessary to see whether statistically significant effects remain or whether trends towards significance would become significant. Secondly, the effectiveness of the combination is likely to be influenced by the timing of each treatment. Since patients were part of our larger comparative trial, patients began VRT at differing intervals. While most began after the three-month CBT follow-up assessment, some began after longer-term follow-ups, which may explain the lessening of the effects of CBT in time. Keeping in mind personalized treatment, patients commenced VRT when they felt CBT treatment gains were consolidated and they were ready to undertake the emotion-inducing sessions of VRT. Third, we did not have a control group consisting of the inverse sequence, that is patients first following VRT and then CBT. However, as noted in patients’ interviews, they found the sequence of CBT followed by VRT to be more complimentary as they gained insight into their illness and then could put into practice their knowledge within the VR dialogue. Additionally, we did not have any patients in the trial desiring to follow CBT after their VRT treatment. The lack of control group was fulfilled with our results from our comparative trial on the individual interventions [45]. 

## 5. Conclusions

To conclude, despite the small sample of the study, we nonetheless found preliminary promising results suggesting the usefulness of merging CBT followed by VRT. The effects of sequencing treatments in this manner may be more than additive since each would enhance the effectiveness of the other. This was observed notably for depressive symptoms and symptoms of schizophrenia. Moreover, these interventions derive from common rationale and are viewed as complimentary with each having their specific therapeutic targets. Firstly, by beginning with CBT, patients may learn to establish links between their thoughts, feelings, or actions with respect to their symptoms and the accompanying dysfunctions [69]. CBT is therefore considered a more op-down approach with its goal aimed to address problematic thinking patterns and core beliefs that may contribute to emotional distress and maladaptive behaviors. CBT for voices thus relies on a considerable degree of abstract self-reflection [70]. In sum, it is aimed at normalizing the psychotic experience, providing a range of alternative explanations, developing a shared understanding of the voices, changing the appraisal of the voices, testing unhelpful beliefs, reducing unhelpful coping strategies and increasing good coping strategies (i.e., mindfulness) [31]. The approach could be enhanced when followed by an experiential psychotherapy, such as VRT, to maximize its effectiveness. VRT uses additional methods for working with voices within the wider context of one’s view of themselves, of relationships with others/voices, and self-narratives [22]. Additionally, the visualization of the avatar/the voice may facilitate the process of validating the experience and modifying the flow of dialogue with the voice through sessions, while altering the voice-hearer relationship [69]. As an experiential therapy, VRT primarily focuses on how patients relate with their voices by targeting self-esteem, emotion regulation and acceptance rather than challenging beliefs about voices. In this vein, this approach is thus considered as a more bottom-up approach. VRT may also allow patients to test/challenge coping mechanism directly in the VR environment with their avatar, while being encouraged to try new strategies throughout the therapy. Combining treatment components in this way and specific order may not only engage multiple treatment targets and possible pathogenic mechanisms but is also likely to have a synergistic effect through the interaction of the individual treatment components [19]. This was confirmed with the interviews held with patients. Particularly for the combination of CBT with VRT, patients may first gain more effective top-down regulation mechanisms with CBT and then they may improve their bottom-up mechanisms with VRT. VRT highlights the future of patient-tailored approaches that integrates several processes relevant to potentially improve the effectiveness of CBT for voices. Future research should nevertheless aim to develop the best merged approach to combine CBT and VRT.

## Figures and Tables

**Table 1:** Sociodemographic and clinical characteristics of the sample.

Sociodemographic		
	Number of participants or Mean	Percentage of participants (%) or Standard deviation (SD)
Gender		
Male	8	(80%)
Female	2	(20%)
Age	43.4	(14.6)
Ethnicity		
Caucasian	10	(100%)
Civil status		
Single	9	(90%)
Couple	1	(10%)
Level of schooling (years)	11.2	(2.7)
**Clinical**		
	Number of participants or Mean	Percentage of participants (%) or Standard deviation (SD)
Diagnosis		
Schizophrenia	8	(80%)
Schizoaffective disorder	2	(20%)
Duration of illness (years)	16.4	(10.9)
Antipsychotic medication		
Atypical	10	(100%)
Clozapine	7	(70%)

**Table 2:** Primary and secondary outcomes throughout time points for cognitive behavioral therapy (CBT) + virtual reality-assisted therapy (VRT).

Mean and Standard Deviation (SD)										Time Points Comparisons						All Time Points	Effect Size					
**T1**		**T2**		**T3**		**T4**		**T5**		***p*** **-Value**						***p*** **-Value**	**T1-T2**	**T1-T3**	**T1-T4**	**T1-T5**	**T3-T4**	**T3-T5**
**Mean**	**SD**	**Mean**	**SD**	**Mean**	**SD**	**Mean**	**SD**	**Mean**	**SD**	**T2-T1**	**T3-T1**	**T4-T1**	**T5-T1**	**T4-T3**	**T5-T3**							
**PSYRATS-AH-Total**																						
31.9	5.1	27.2	11.2	28.1	11.5	24.5	11.4	19.8	15.6	0.148	0.235	0.026	0.001	0.271	0.019	0.015	0.540	0.427	0.838	1.043	0.314	0.606
**PSYRATS-AH-Distress**																						
15.3	4.5	12.4	5.3	13.6	6.5	12.4	6.4	9.8	7.4	0.119	0.360	0.119	0.007	0.507	0.053	0.088	0.590	0.304	0.524	0.898	0.186	0.546
**PSYRATS-AH-Frequency**																						
8.0	2.9	7.2	3.4	7.4	3.2	6.0	2.7	4.9	4.2	0.347	0.456	0.022	0.001	0.112	0.008	0.010	0.253	0.196	0.714	0.859	0.473	0.670
**PSYRATS-AH-Attribution**																						
6.7	1.2	5.4	2.2	5.3	2.1	4.9	2.6	4.1	3.4	0.075	0.050	0.015	0.001	0.608	0.111	0.019	0.734	0.819	0.889	1.020	0.169	0.425
**PSYRATS-AH-Loudness**																						
1.9	1.0	2.2	1.2	1.8	1.1	1.2	0.6	1.0	0.9	0.477	0.843	0.102	0.050	0.148	0.074	0.047	0.272	0.095	0.849	0.946	0.677	0.796
**BAVQ-R-Total**																						
51.3	16.0	47.0	16.2	45.0	14.9	43.5	17.8	50.6	10.7	0.239	0.089	0.037	0.964	0.677	0.139	0.160	0.267	0.408	0.461	0.051	0.091	0.432
**BAVQ-R-Persecutory beliefs**																						
15.5	6.6	12.0	5.8	12.6	5.7	12.0	6.0	14.6	4.2	0.049	0.097	0.049	0.619	0.740	0.316	0.182	0.563	0.470	0.555	0.163	0.103	0.399
**BAVQ-R-Benevolence**																						
3.2	5.4	3.3	4.4	2.8	3.7	3.5	5.1	3.1	3.0	0.869	0.492	0.620	0.570	0.240	0.206	0.706	0.020	0.086	0.057	0.023	0.157	0.089
**BAVQ-R-Engagement**																						
4.2	6.0	3.7	4.9	2.4	4.2	3.8	5.8	3.1	4.4	0.325	0.001	0.430	0.409	0.010	0.028	0.016	0.091	0.348	0.068	0.209	0.276	0.163
**BAVQ-R-Resistance**																						
19.8	8.2	18.3	5.9	18.9	3.1	18.8	4.5	20.5	4.0	0.326	0.554	0.516	0.919	0.938	0.530	0.815	0.210	0.145	0.151	0.109	0.026	0.447
**BDI-II-Total**																						
23.6	12.4	15.0	8.6	17.9	12.0	14.2	11.6	11.2	11.9	0.002	0.030	0.004	<0.001	0.371	0.015	0.001	0.806	0.467	0.783	1.020	0.314	0.561
**PANSS-Total**																						
76.0	7.5	73.6	13.6	75.8	18.4	64.4	15.5	67.1	13.7	0.515	0.958	0.003	0.088	0.003	0.097	0.013	0.320	0.014	0.953	0.806	0.670	0.536
**PANSS-Positive**																						
14.8	1.9	13.4	4.7	13.9	4.1	12.1	2.8	12.6	3.6	0.150	0.368	0.007	0.068	0.062	0.309	0.079	0.391	0.282	1.128	0.764	0.513	0.337
**PANSS-Negative**																						
14.2	2.6	14.0	4.5	14.5	5.0	11.8	5.5	12.9	4.5	0.863	0.796	0.044	0.465	0.024	0.333	0.159	0.054	0.075	0.558	0.354	0.514	0.336
**PANSS-Disorganized**																						
8.1	2.2	8.2	2.7	7.3	3.1	6.9	2.2	6.1	1.6	0.854	0.135	0.032	0.013	0.491	0.241	0.022	0.041	0.298	0.545	1.040	0.149	0.486
**PANSS-Excited/Hostility**																						
6.7	1.8	7.1	1.7	6.8	2.1	5.2	1.3	5.5	1.8	0.504	0.823	0.016	0.101	0.009	0.066	0.011	0.228	0.051	0.955	0.667	0.916	0.665
**PANSS-Anxio-depressive/affective**																						
9.0	2.4	8.7	2.8	9.3	2.9	8.4	3.1	8.2	2.4	0.675	0.642	0.404	0.295	0.197	0.142	0.558	0.115	0.113	0.216	0.333	0.300	0.413
**Q-LES-Q-SF**																						
52.6	6.5	54.4	6.7	52.1	5.3	54.6	5.3	54.7	7.8	0.206	0.740	0.161	0.086	0.086	0.045	0.162	0.273	0.084	0.337	0.331	0.472	0.451

Data are raw mean score with standard deviation (SD). Linear mixed models with maximum-likelihood estimation were used. Absolute values for effect sizes are reported. Time points: T1 = before CBT, T2 = after CBT, T3 = follow-up CBT/baseline VRT, T4 = post VRT, T5 = three-month follow-up VRT. Abbreviations: PSYRATS–AH = Psychotic Symptoms Rating Scale—Auditory Hallucinations; BAVQR = Beliefs About Voices Questionnaire; BDI-II = Beck Depression Inventory-II; PANSS = Positive and Negative Symptom Scale; Q-LES-Q-SF = Quality of Life Enjoyment and Satisfaction Questionnaire—Short From.
